# Insular glioma and emotional states affect the whole brain network: a task-state electroencephalography study

**DOI:** 10.3389/fneur.2026.1790718

**Published:** 2026-04-17

**Authors:** Chuanhao Zhang, Zuocheng Yang, Qifeng He, Hongfang Zhao, Xinxin Wang, Zhenghai Deng, Ruquan Han, Zonggang Hou, Jian Xie

**Affiliations:** 1 Department of Neurosurgery, Beijing Tiantan Hospital, Capital Medical University, Beijing, China; 2 Department of Neurosurgery, Beijing Hospital, National Center for Gerontology, National Clinical Research Center for Gerontology, The Key Laboratory of Geriatrics of NHC, Institute of Geriatric Medicine, Chinese Academy of Medical Sciences, Beijing, China; 3 Department of Anesthesiology, Beijing Tiantan Hospital, Capital Medical University, Beijing, China; 4 China National Clinical Research Center for Neurological Diseases, Beijing, China

**Keywords:** brain networks, emotion, glioma, insular, task-state electroencephalography

## Abstract

**Purpose:**

This study investigated the effects of insular gliomas on brain functional network dynamics during auditory stimulus processing via task-state electroencephalography (EEG) combined with graph theory analysis.

**Methods:**

Thirty-three insular glioma patients (PT) and seventeen healthy controls (HC) underwent auditory Oddball task-based EEG recording. Graph theory analysis was used to assess functional connectivity and core network indices (Cp, locE, gE, Lp, *σ*) across four frequency bands. Neuropsychological assessments (MoCA, MMSE, HAMA, HDRS) were performed to evaluate cognitive and emotional states.

**Results:**

PT and HC had no significant demographic differences, but PT showed lower MoCA/MMSE scores and higher HAMA/HDRS scores (all p0.05 for left). Abnormal beta band functional connections between PT and HC were more extensive under novel (342) than standard stimuli (220); left and right insular glioma patients had 764 and 438 abnormal beta-band connections under novel stimuli, respectively. PT had significantly lower beta band Cp, locE and small-worldness coefficient under novel stimuli than HC, with such reductions only significant in right insular glioma patients. These beta band indices in PT were negatively correlated with HAMA/HDRS scores under novel stimuli, and this correlation was specific to right insular glioma patients (all *p* > 0.05 for left).

**Conclusion:**

Insular gliomas impair beta band whole brain functional network integration and small-worldness under novel stimuli, with prominent hemispheric specificity: right insular glioma patients show significant topological indicator damage, while left insular glioma patients maintain stable networks via left dominant hemisphere interhemispheric compensatory plasticity. Beta band Cp, locE and small-worldness coefficient under novel stimuli are potential neurophysiological indicators for insular glioma assessment, especially for right insular lesions. These indices are closely associated with anxiety and depression in right insular glioma patients, highlighting the need to consider tumor laterality in clinical evaluation and implement comprehensive emotional interventions for such patients.

## 1 Introduction

The insula, a critical region within the brain, is deeply involved in advanced brain functions such as emotion regulation, cognitive processes, and sensory information processing (1, 2). Notably, the insula constitutes a preferential location for gliomagenesis, harboring 25% of low-grade gliomas and 10.8% of supratentorial glioblastomas (GBMs) (3). Due to its unique anatomical location, insular gliomas not only potentially disrupt the normal functioning of the insula but may also exert profound effects on widespread brain regions through complex neural network connections, thereby altering patients’ emotional experiences, cognitive abilities, and behavioral manifestations (4, 5). In recent years, with the rapid advancement of neuroimaging and electrophysiological techniques, researchers have begun to delve deeply into how insular gliomas influence patients’ brain functional networks, as well as their cognitive and emotional states (6, 7).

As a key neuroimaging modality, functional magnetic resonance imaging (fMRI) indirectly infers neuronal activity via detecting blood oxygenation level changes during brain function (8, 9). It has been extensively applied in constructing brain functional networks. Within the realm of insular glioma research, fMRI technology, leveraging its high spatial resolution, has successfully elucidated the functional connectivity patterns between the insula and other brain regions, along with alterations in these connections under pathological conditions (10–12). However, the relatively low temporal resolution of fMRI poses challenges in precisely capturing real-time dynamic changes in the brain during rapid cognitive tasks, thereby limiting its comprehensive understanding of the dynamic properties of brain functional networks (5).

Electroencephalogram (EEG) is a neurophysiological detection technique that records the rhythmic electrical activity spontaneously generated by a group of brain neurons in a resting or active state through scalp electrodes, and presents it in waveform form. Its core principle lies in capturing the weak electrical signals generated by the synchronous firing of cortical neurons, thereby reflecting the dynamic alterations in brain functional states and neural activity. Task-State Electroencephalogram (Task-EEG) refers to the real-time recording of EEG signal patterns formed by the electrical activity of a group of brain neurons through scalp electrodes during the active execution of specific cognitive, motor, or perceptual tasks by a subject. It is used to analyze the dynamic response mechanism and functional localization of the brain to task stimuli. Compared with fMRI and DTI techniques, task state electroencephalography, as a non-invasive and efficient neurophysiological recording technique, has extremely high temporal resolution and can capture real-time brain activity during specific cognitive tasks (13–15). This provides a robust tool for investigating dynamic changes in brain functional networks. Through the design of classic cognitive tasks such as the Oddball paradigm, task-state EEG studies can precisely elicit brain responses to various types of stimuli (standard, deviant, and novel stimuli), facilitating the analysis of underlying neural mechanisms and their associations with cognitive functions and emotional states.

This study focuses on the patient population with insular gliomas, employing task-state EEG technology in conjunction with graph-theoretic analysis methods. Our aim is to comprehensively reveal the impact of insular gliomas on patients’ brain functional networks across different frequency bands, particularly their dynamic network properties during the processing of various auditory stimuli. By comparing with a healthy control group, we aspire to clarify the alterations in brain network connectivity patterns induced by insular gliomas and how these changes correlate with patients’ cognitive impairments and abnormal emotional states, thereby providing theoretical support for the identification of disease states and treatment of insular glioma.

## 2 Materials and methods

### 2.1 Study population

A total of 33 patients with insular gliomas were recruited from Beijing Tiantan Hospital between 2022 and 2024. All were newly diagnosed with unilateral insular gliomas, treatment-naive to adjuvant oncological therapies, and free of other neurological or psychiatric disorders. Clinical and demographic data were obtained from the institutional medical records. In addition, this study recruited 17 healthy controls (HC) and required them to complete questionnaires on basic information and health status. Based on the answers, individuals with chronic disease risk, drug dependence, history of mental illness, cardiovascular disease, hypertension, stroke and hearing impairment were excluded. All PT and HC were confirmed to be right-handed using the Edinburgh Handedness Inventory. This study was approved by the Institutional Review Board of Beijing Tiantan Hospital, and written informed consent was obtained from all participants prior to enrollment.

The inclusion criteria of patients are: newly diagnosed with insular glioma (WHO grade 2–4), with the lesion predominantly located in the insular cortex; aged 18–55 years; unilateral insular glioma; native Chinese speaker; voluntary participation and ability to comply with the study schedule and testing procedures; no epileptic seizures within 1 month prior to performing the oddball task.

The exclusion criteria of patients are: presence of other neurological or psychiatric disorders; severe systemic diseases, including infectious diseases, hypertension, and diabetes mellitus; prior receipt of adjuvant oncological therapies; hearing impairment; history of long-term smoking, alcohol consumption, or use of psychotropic medications; left-handedness.

### 2.2 EEG recording and pre-processing

EEG signals were recorded using a 64-channel Ag-AgCl electrode cap (BrainAmpMR, Brain Products GmbH, Germany). Signals were digitized online at a sampling rate of 1,000 Hz, and the resistance was controlled below 20 k*Ω* for each electrode (most were below 10 K Ω). CPz was set as a reference electrode during recording.

Auditory stimuli consisted of three types: standard stimuli (Std)-500 Hz pure tones with 100 ms duration and 70% occurrence probability; deviant stimuli (Dev)- 1000 Hz pure tones with 100 ms duration and 15% probability; and novel stimuli (Nov)-computer-generated recordings of each patient’s own name, also presented at 15% probability. A total of 400 stimuli were delivered in a pseudorandom order using E-Prime 3.0 software (Psychology Software Tools, Pittsburgh, PA), with interstimulus intervals ranging from 800 to 1,200 ms, resulting in an approximate task duration of 8 min. To ensure stimulus salience, deviant and novel stimuli were each preceded by at least two consecutive standard tones. Auditory stimulation was delivered binaurally via Sennheiser CX 80S earphones, with volume adjusted to a comfortable level for each participant (range: 60–80 dB).

Raw EEG data were preprocessed using the EEGLAB toolbox within the MATLAB environment (version R2022b). Continuous EEG signals were band-pass filtered at 0.1–80 Hz. Data were segmented according to stimulus type, and baseline correction was performed using the pre-stimulus interval. Segmented epochs were subsequently visually inspected, and independent component analysis (ICA) was applied to remove artifacts caused by eye blinks and body movements. Finally, average values of the bilateral mastoids were used for re-referencing.

The specific criteria for excluding independent components during ICA (Independent Component Analysis) are as follows: First, identify and exclude independent components associated with eye movements (like blinks, saccades). These components typically exhibit the following characteristics: The component waveform demonstrates a typical blink or saccade pattern, such as short-duration, high-amplitude pulse-like waveforms. Energy is predominantly concentrated in the low-frequency range (<4 Hz), consistent with the spectral properties of eye movement artifacts. The spatial distribution of the component shows a concentration in the prefrontal or orbital regions, aligning with the scalp projection of eye movement artifacts. Second, exclude independent components related to muscular activity (like chewing, swallowing, facial muscle contractions). The features of these components include: The component waveform exhibits irregular, high-frequency fluctuations, consistent with the rapid changes in electromyographic (EMG) activity. Energy is primarily concentrated in the high-frequency range (>20 Hz), particularly within the beta (13–30 Hz) frequency bands. The spatial distribution of the component shows localized concentration, such as in the temporal or jaw-facial regions, consistent with the anatomical locations of muscle activity. Third, identify and exclude independent components associated with cardiac activity. The characteristics of these components include: The component waveform displays periodic, spike-like pulses, resembling the morphology of QRS complexes in electrocardiographic activity. Power was predominantly concentrated in the low-frequency range (<10 Hz), consistent with the spectral characteristics of cardiac activity. The spatial distribution of this component typically showed a widespread pattern or focal clustering over specific channels. Fourth, exclude noise components resulting from poor electrode contact or external interference. The features of these components include: The component waveform exhibits random, irregular fluctuations without clear physiological significance. Energy is uniformly distributed without concentration in any specific frequency band. The spatial distribution of the component may be limited to a single or a few leads, with abnormally high amplitudes. Last but not the least, exclude residual components that cannot be classified as neurophysiological activity or clear artifacts. These components typically lack distinct time-domain or frequency-domain features and exhibit widespread but non-concentrated scalp distributions.

### 2.3 Source localization

EEG source localization analysis was performed using the Brainstorm toolbox (version 3.2.1.0). Spatial normalization was implemented using a standardized head model derived from the Montreal Neurological Institute (MNI) Colin27 template. The forward problem was computed via the OpenMEEG boundary element method (BEM), which characterizes head volume conduction across three conductive tissue layers: scalp, skull, and brain (16). For the inverse problem, cortical dipole activities were reconstructed using the weighted minimum norm estimation (wMNE) algorithm (17). This approach constrained dipole orientations to the cortical surface normal vectors and incorporated depth weighting to mitigate bias toward superficial sources. A total of 15,002 cortical dipole sources were uniformly distributed across the gray matter surface, with regularization parameter *λ* set to 1/signal-to-noise ratio (SNR)^2^ for optimal source reconstruction.

In this study, cortical parcels were delineated using the Desikan-Killiany (DK) atlas, a widely validated neuroanatomical template integrated in FreeSurfer (18). This atlas employs macroscopic gyral landmarks to partition the cerebral cortex into 68 anatomically homogeneous regions, with each parcel assigned to one of seven major cortical systems: frontal cortex, temporal cortex, parietal cortex, occipital cortex, insular cortex, central cortex, and limbic cortex.

In the study of brain networks, two fundamental components are systematically defined: nodes represent anatomically delineated brain regions, while edges quantify functional or structural connections between these regions. For this investigation, the DK atlas was employed to parcellate the cerebral cortex into 68 nodes, each corresponding to a distinct macroanatomical region. Time series of cortical sources at each of the 68 nodes were band-pass filtered and segmented into four frequency bands: delta (1–4 Hz), theta (4–8 Hz), alpha (8–13 Hz), and beta (13–30 Hz).

### 2.4 Connectivity and network analysis

The Phase Lag Index (PLI) was adopted to quantify phase synchronization between neuroelectric time series. As a robust metric of functional connectivity, PLI ranges from 0 to 1: a value of 0 signifies absent coupling or a symmetric phase-difference distribution around zero, while a value of 1 denotes perfect phase synchronization. This index is particularly advantageous for electrophysiological data analysis as it minimizes the influence of volume conduction and common reference artifacts, focusing exclusively on consistent phase relationships. PLI values were calculated for all pairwise combinations of the 68 brain regions across the delta, theta, alpha and beta four frequency bands and the standard, deviant and novel three stimulus conditions. All PLI values were retained for subsequent graph theory analysis, no predefined weight thresholds were applied to filter low PLI values to retain the full spectrum of functional connectivity information, avoiding potential loss of weak but biologically meaningful connections that might be relevant to the brain network alterations induced by insular gliomas. In the present investigation, PLI served as the primary measure of functional connectivity, with higher values indicating stronger phase synchronization between channel pairs. The computational methodology for PLI has been comprehensively described in prior literature (19). We calculated and compared PLI in delta, theta, alpha and beta frequency bands of the two groups (PT and HC) under standard, deviant and novel stimulis, respectively, and connectivities with significant differences were extracted by the independent t-tests (*p* < 0.05).

Quantitative analyses of the weighted network were performed using graph theory. Five global network metrics were assessed as follows: (1) Characteristic path length (Lp): the mean shortest path length across all node pairs within the network; (2) Global efficiency (gE): the average of the reciprocal shortest path lengths between all node pairs; (3) Local efficiency (locE): measuring the efficiency of information transmission between node neighbors, reflecting the fault tolerance and local information integration capability of the network; (4) Clustering coefficient (Cp): the ratio of actual existing connections among the neighbors of a node to the total number of potential connections between those neighboring nodes; (5) Small-worldness coefficient (*σ*): the normalized ratio of clustering coefficient to characteristic path length quantifies whether the network has both high clustering coefficient and short characteristic path length (20, 21). And then conducted group-level statistical tests.

### 2.5 Neurocognitive function test

All participants underwent a comprehensive neuropsychological assessment battery, including the Montreal Cognitive Assessment (MoCA), Mini-Mental State Examination (MMSE), Hamilton Anxiety Rating Scale (HAMA), and Hamilton Depression Rating Scale (HDRS). All assessments were administered in a quiet, controlled environment to minimize external distractions and ensure reliable results.

The MoCA scale is a well-validated tool specifically designed for the rapid screening of mild cognitive impairment and early-stage Alzheimer’s disease (22). It assesses multiple cognitive domains, including attention, language, memory, visuospatial abilities, executive function, calculation, orientation, and abstract reasoning. Each assessment took approximately 10–15 min, with minor variation depending on individual response speed and cognitive processing capacity. The total score of the MoCA ranges from 0 to 30 points; scores below 26 (adjusted for educational level) are typically considered suggestive of mild cognitive impairment. Notably, one point was added to the raw scores of participants with fewer than 12 years of formal education to account for the potential influence of educational background on cognitive test performance.

We employed the Hamilton Anxiety Rating Scale (HAMA) and the Hamilton Depression Rating Scale (HDRS) as assessment tools to measure patients’ levels of anxiety and depression (23, 24). The Hamilton Anxiety Scale (HAMA) consists of 14 items, and each item is rated based on the severity of symptoms, ranging from 0 (indicating no symptoms) to 4 (indicating severe symptoms). The total score provides a comprehensive reflection of overall anxiety severity, with higher scores indicating more severe anxiety symptoms. The Hamilton Depression Rating Scale (HDRS) evaluates multiple dimensions of depression, including depressed mood, insomnia, diminished interest in work and activities, and other related symptoms. Consistent with the HAMA, all items are scored based on symptom severity, and a higher total score indicates more severe depressive symptoms.

The Mini-Mental State Examination (MMSE) assesses multiple cognitive domains, including attention and calculation, memory, language, visuospatial function, and orientation (25). The total score of the MMSE ranges from 0 to 30 points, with the following scoring criteria for cognitive status: 27–30 points indicate normal cognition, scores below 27 points suggest cognitive impairment (further categorized as 21–26 points for mild cognitive impairment, 10–20 points for moderate cognitive impairment, and 0–9 points for severe cognitive impairment).

### 2.6 Statistical analysis

The EEG source localization analysis was conducted using the Brainstorm toolbox (version 3.2.1.0). A standardized head model based on the Montreal Neurological Institute (MNI) Colin27 template was employed for spatial normalization. We used the GRETNA package to calculate PLI and graph theory indicators. FDR correction was used for repeated measurement statistical analysis, with *p* value less than 0.05 was considered significant.

Statistical analyses were conducted using SPSS version 23.0 (IBM Corp., Armonk, NY, United States). For continuous variables, group comparisons were performed using independent-samples *t*-tests. If data departed significantly from a normal distribution (as determined by Shapiro–Wilk tests or graphical inspection), nonparametric Mann–Whitney U tests were applied instead. Correlations between neurophysiological metrics and neuropsychological test scores were examined using Spearman’s rank correlation. All statistical tests were two-sided, and a *p*-value < 0.05 was deemed statistically significant.

## 3 Results

### 3.1 Clinical and demographic characteristics

A total of 33 patients with insular glioma were enrolled from Beijing Tiantan Hospital between 2022 and 2024, along with 17 healthy control subjects. Of these patients, 15 harbored left-sided insular gliomas and 18 had right-sided lesions, with all patients demonstrating unilateral involvement of the entire insular lobe. No significant intergroup differences were observed in age, sex, BMI, or educational level (all *p* > 0.05) (Table 1). Comparisons of neuropsychological scale scores between patients with insular glioma and healthy controls are presented in Table 2.

**Table 1:** Clinical characteristics and demographic features of participants.

Groups	Total numbers	Sex (male)	Age	BMI	Education level (year)	IDH mutation status (mutation)	P value (compared with HC)
Patients	33	20	42.27 ± 1.43	24.50 ± 0.69	13.1 ± 0.89	26	0.78 (Sex); 0.09 (Age); 0.53 (BMI); 0.77 (Education level)
Left insular glioma patients	15	9	43.27 ± 1.99	24.63 ± 0.71	13.56 ± 1.28	10	0.99 (Sex); 0.07 (Age); 0.45 (BMI); 0.53 (Education level)
Right insular glioma patients	18	11	41.44 ± 2.06	24.38 ± 1.13	12.75 ± 1.27	16	1.00 (Sex); 0.22 (Age); 0.70 (BMI); 0.99 (Education level)
Healthy controls	17	11	37.76 ± 0.74	23.85 ± 0.74	12.76 ± 0.60	NA	NA

**Table 2:** The comparison of neuropsychological scales scores among participants.

Groups	MOCA	MMSE	HDRS	HAMA
Healthy controls	29.82 ± 0.10	29.88 ± 0.08	0.06 ± 0.06	0.06 ± 0.06
Patients	23.24 ± 1.15	27.50 ± 0.93	10.19 ± 2.20	6.19 ± 1.75
*P* value (PT vs. HC)	<0.01**	0.02*	<0.01*	0.002**
Left patients	22.67 ± 1.74	28.14 ± 0.77	11.78 ± 3.53	6.22 ± 1.98
*P* value (Left-PT vs. HC)	0.003**	0.04*	0.01*	<0.01**
Right patients	23.67 ± 1.60	27.10 ± 1.47	9.00 ± 2.87	6.17 ± 2.75
*P* value (Right-PT vs. HC)	0.003**	0.025*	0.01*	0.048*

*p < 0.05, **p < 0.01. Healthy control group vs. group of glioma patients on the whole insula/left insula/right insula.

### 3.2 Functional connectivity and graph analysis

To investigate the regulatory role of tumor lateralization in the topological structure of brain networks, patients with insular glioma were stratified into the left insular glioma group (*n* = 15) and the right insular glioma group (*n* = 18) according to tumor lateralization for stratified comparative analysis with the HC group. Meanwhile, the PT group (*n* = 33) was used as an overall control to systematically evaluate the specific effect of tumor lateralization on abnormalities in brain network functional connectivity and topological metrics.

**Figure 1:**
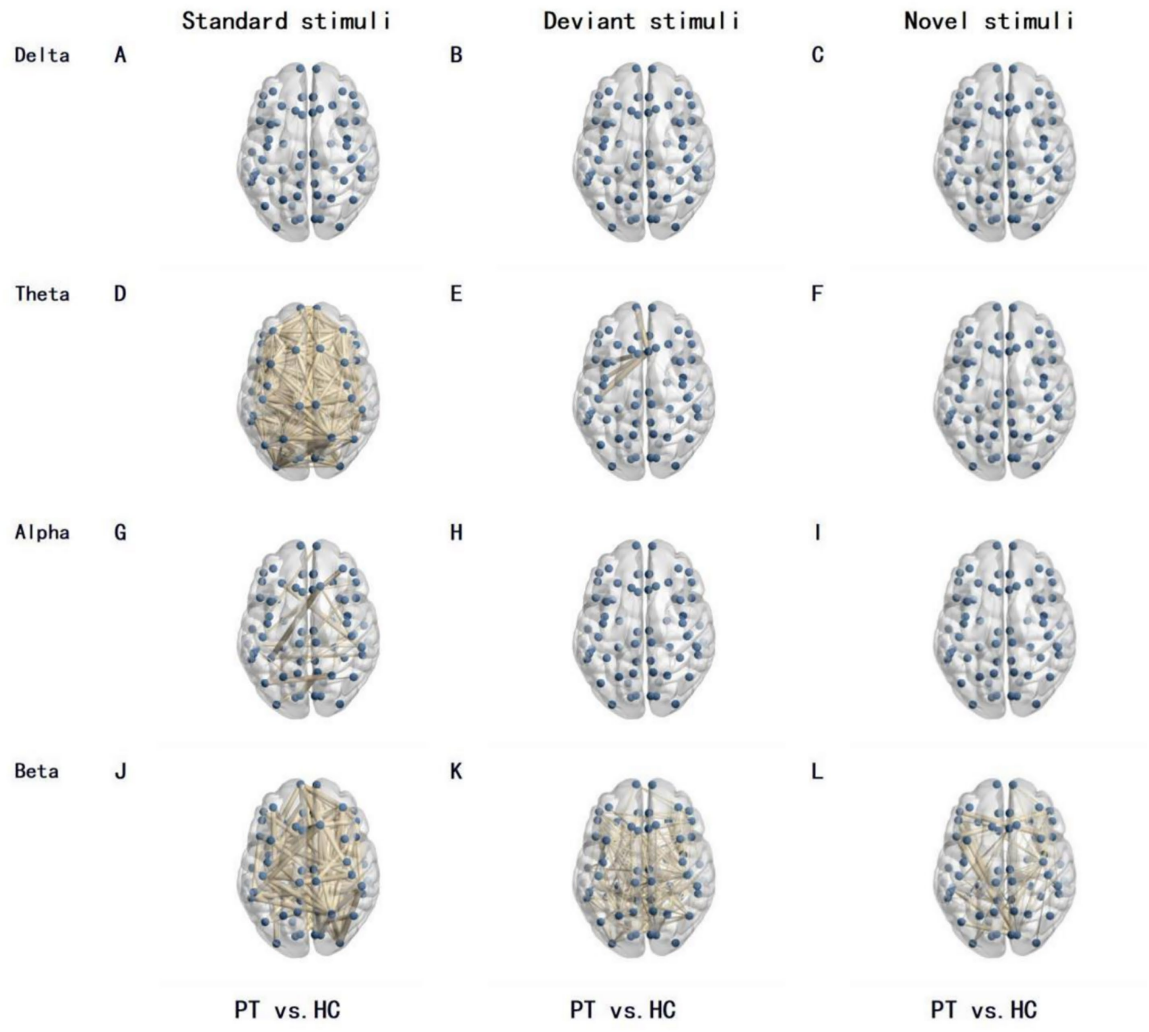
Differences in brain network functional connectivity between PT and the HC under different frequency bands and stimulation types. The lines in the figure represent functional connections with significant differences, with thicker lines indicating greater differences in functional connections. The number of lines represents the total number of functional connections with differences between the two groups. **(A–C)** Delta band, for standard, deviant, and novel stimuli, respectively; **(D–F)** Theta band, for standard, deviant, and novel stimuli, respectively; **(G–I)** Alpha band, for standard, deviant, and novel stimuli, respectively; **(J–L)** Beta band, for standard, deviant, and novel stimuli, respectively.

**Figure 2:**
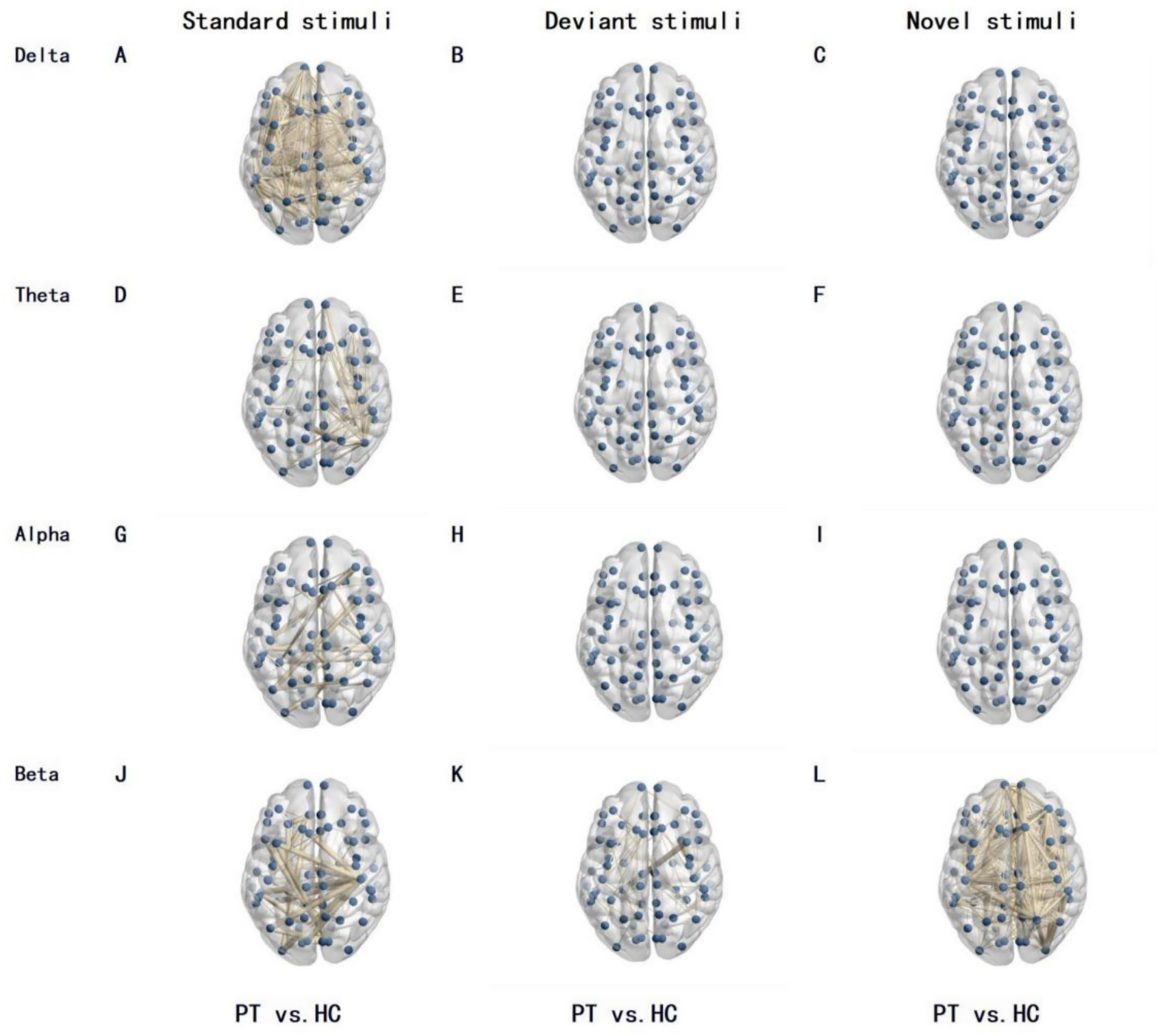
Differences in brain network functional connectivity between left insular glioma patients and the HC under different frequency bands and stimulation types. The lines in the figure represent functional connections with significant differences, with thicker lines indicating greater differences in functional connections. The number of lines represents the total number of functional connections with differences between the two groups. **(A–C)** Delta–band, for standard, deviant, and novel stimuli, respectively; **(D–F)** theta band, for standard, deviant, and novel stimuli, respectively; **(G–I)** alpha band, for standard, deviant, and novel stimuli, respectively; **(J–L)** beta band, for standard, deviant, and novel stimuli, respectively.

**Figure 3:**
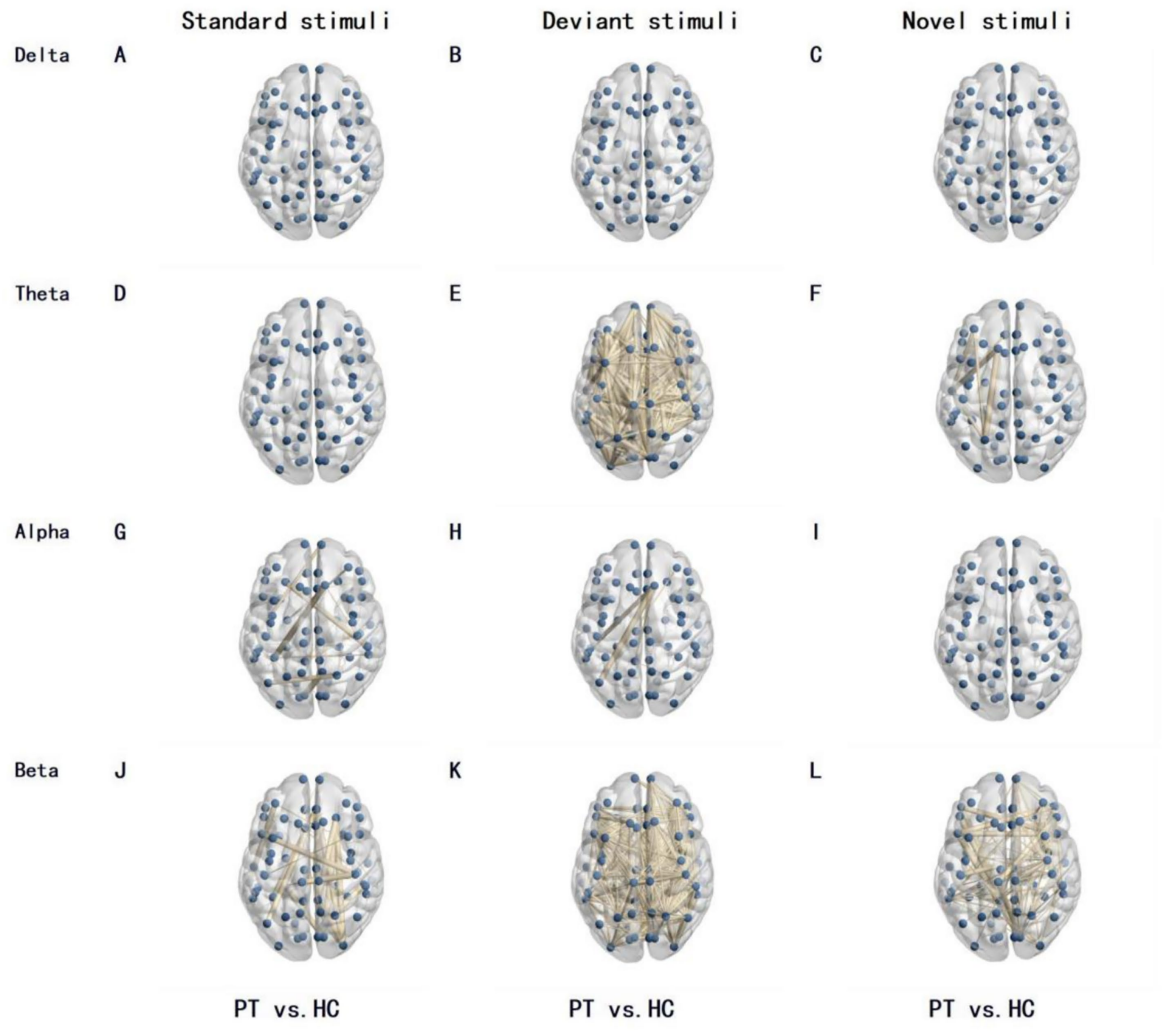
Differences in brain network functional connectivity between right insular glioma patients and the HC under different frequency bands and stimulation types. The lines in the figure represent functional connections with significant differences, with thicker lines indicating greater differences in functional connections. The number of lines represents the total number of functional connections with differences between the two groups. **(A–C)** Delta band, for standard, deviant, and novel stimuli, respectively; **(D–F)** Theta band, for standard, deviant, and novel stimuli, respectively; **(G–I)** Alpha band, for standard, deviant, and novel stimuli, respectively; **(J–L)** Beta band, for standard, deviant, and novel stimuli, respectively.

Differences in functional connectivity between PT and HC group were mainly concentrated in the beta band, and were jointly regulated by stimulus type and tumor lateralization: 220 differentially functional connections were observed between PT and HC group under standard stimuli in the beta band, increasing to 342 under novel stimuli; only 116 differentially functional connections were found between the left insular glioma group and the HC group under standard stimuli in the beta band, surging to 764 under novel stimuli; merely 18 differentially functional connections were identified between the right insular glioma group and the HC group under standard stimuli in the beta band, with 438 under novel stimuli (Figures 1–3).

**Figure 4:**
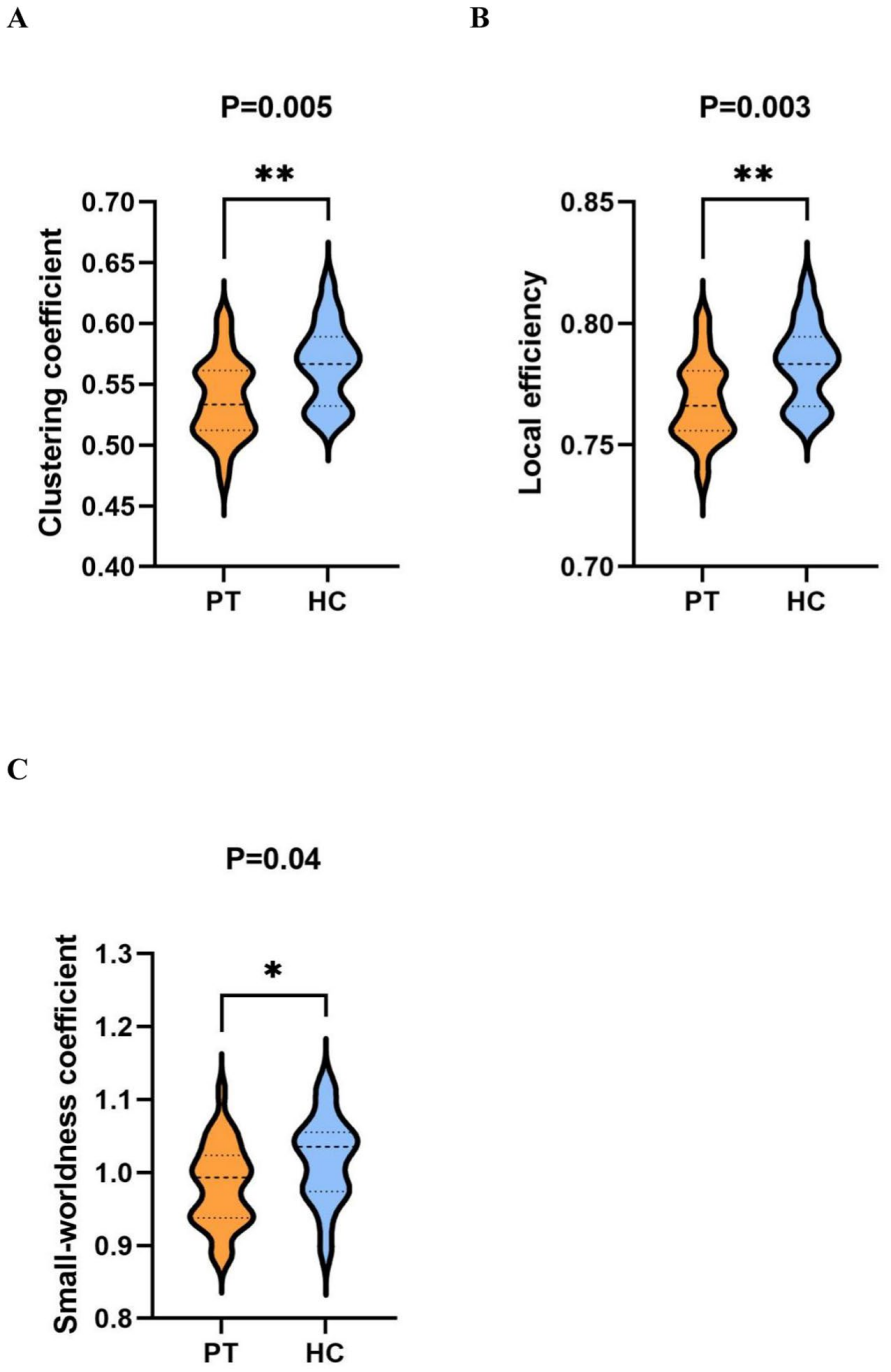
Comparison of graph theory indicators within the beta band under novel stimuli between PT and HC. **(A–C)** are violin plots comparing the clustering coefficient, local efficiency, and smallworldness coefficient between two groups, respectively.

**Figure 5:**
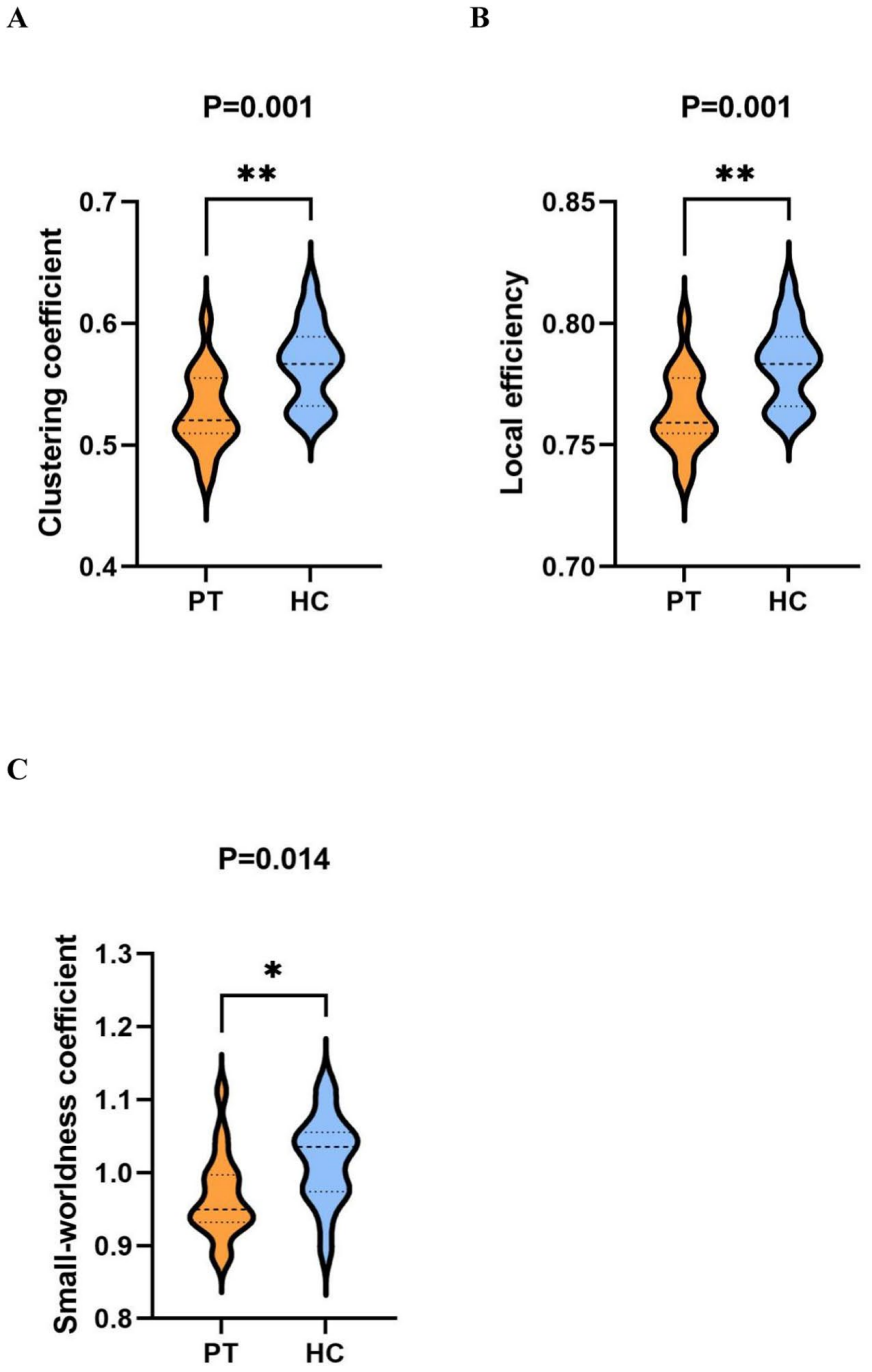
Comparison of graph theory indicators within the beta band under novel stimuli between the right insular glioma patients group and HC. **(A–C)** are violin plots comparing the clustering coefficient, local efficiency, and small-worldness coefficient between two groups, respectively.

Analysis of graph theory metrics revealed a significant hemispheric-specific effect of tumor lateralization on the core brain network metrics under novel stimuli in the beta band: the Cp, locE, and small-worldness coefficient of both the total insular glioma group and the right insular glioma group were significantly lower than those of the HC group (Figures 4, 5), while no statistically significant differences were found in the above graph theory metrics between the left insular glioma group and the HC group. These results suggest that tumor lateralization is a key regulatory factor for abnormalities in the topological structure of brain networks in patients with insular glioma (Table 3).

**Table 3:** The comparison of graph theory indicators between PT and HC groups.

Group	Frequency band	Stimulus type	Lp	GE	LE	CC	σ
PT	Delta	Std	0.47 ± 0.01	0.744 ± 0.01	0.84 ± 0.01	0.68 ± 0.01	1.03 ± 0.02
		Dev	0.47 ± 0.01	0.74 ± 0.01	0.79 ± 0.03	0.63 ± 0.04	0.98 ± 0.05
		Nov	0.39 ± 0.01	0.75 ± 0.01	0.84 ± 0.01	0.68 ± 0.02	1.03 ± 0.03
	Theta	Std	0.69 ± 0.01	0.75 ± 0.01	0.81 ± 0.02	0.64 ± 0.02	0.10 ± 0.03
		Dev	0.64 ± 0.01	0.75 ± 0.01	0.83 ± 0.01	0.65 ± 0.03	1.02 ± 0.03
		Nov	0.59 ± 0.01	0.75 ± 0.01	0.80 ± 0.01	0.61 ± 0.01	1.01 ± 0.03
	Alpha	Std	0.65 ± 0.02	0.74 ± 0.01	0.80 ± 0.02	0.62 ± 0.02	1.03 ± 0.03
		Dev	0.63 ± 0.01	0.74 ± 0.01	0.81 ± 0.01	0.63 ± 0.02	1.04 ± 0.03
		Nov	0.57 ± 0.02	0.75 ± 0.01	0.79 ± 0.02	0.61 ± 0.02	1.02 ± 0.03
	Beta	Std	0.29 ± 0.01	0.76 ± 0.01	0.77 ± 0.01	0.54 ± 0.01	0.97 ± 0.02
		Dev	0.29 ± 0.01	0.76 ± 0.01	0.78 ± 0.01	0.56 ± 0.01	1.01 ± 0.02
		Nov	0.28 ± 0.01	0.76 ± 0.01	0.77 ± 0.01	0.54 ± 0.01	0.98 ± 0.01
HC	Delta	Std	0.49 ± 0.03	0.741 ± 0.01	0.74 ± 0.07	0.59 ± 0.06	0.92 ± 0.09
		Dev	0.46 ± 0.02	0.75 ± 0.01	0.83 ± 0.01	0.66 ± 0.02	1.02 ± 0.03
		Nov	0.37 ± 0.01	0.76 ± 0.01	0.84 ± 0.01	0.69 ± 0.02	1.03 ± 0.01
	Theta	Std	0.67 ± 0.02	0.76 ± 0.01	0.80 ± 0.03	0.63 ± 0.03	0.99 ± 0.04
		Dev	0.64 ± 0.02	0.74 ± 0.01	0.82 ± 0.01	0.64 ± 0.02	1.03 ± 0.03
		Nov	0.58 ± 0.01	0.75 ± 0.01	0.66 ± 0.08	0.50 ± 0.06	0.85 ± 0.10
	Alpha	Std	0.63 ± 0.03	0.743 ± 0.01	0.75 ± 0.05	0.57 ± 0.04	0.94 ± 0.07
		Dev	0.63 ± 0.03	0.75 ± 0.01	0.78 ± 0.03	0.60 ± 0.04	0.98 ± 0.06
		Nov	0.57 ± 0.03	0.75 ± 0.01	0.81 ± 0.01	0.62 ± 0.02	1.018 ± 0.01
	Beta	Std	0.28 ± 0.01	0.759 ± 0.01	0.78 ± 0.01	0.57 ± 0.01	1.01 ± 0.02
		Dev	0.27 ± 0.01	0.76 ± 0.01	0.78 ± 0.01	0.56 ± 0.02	1.00 ± 0.02
		Nov	0.27 ± 0.01	0.76 ± 0.01	0.78 ± 0.01	0.57 ± 0.01	1.02 ± 0.01

### 3.3 Correlational analyses of brain network measures and neuropsychological test performance

**Figure 6:**
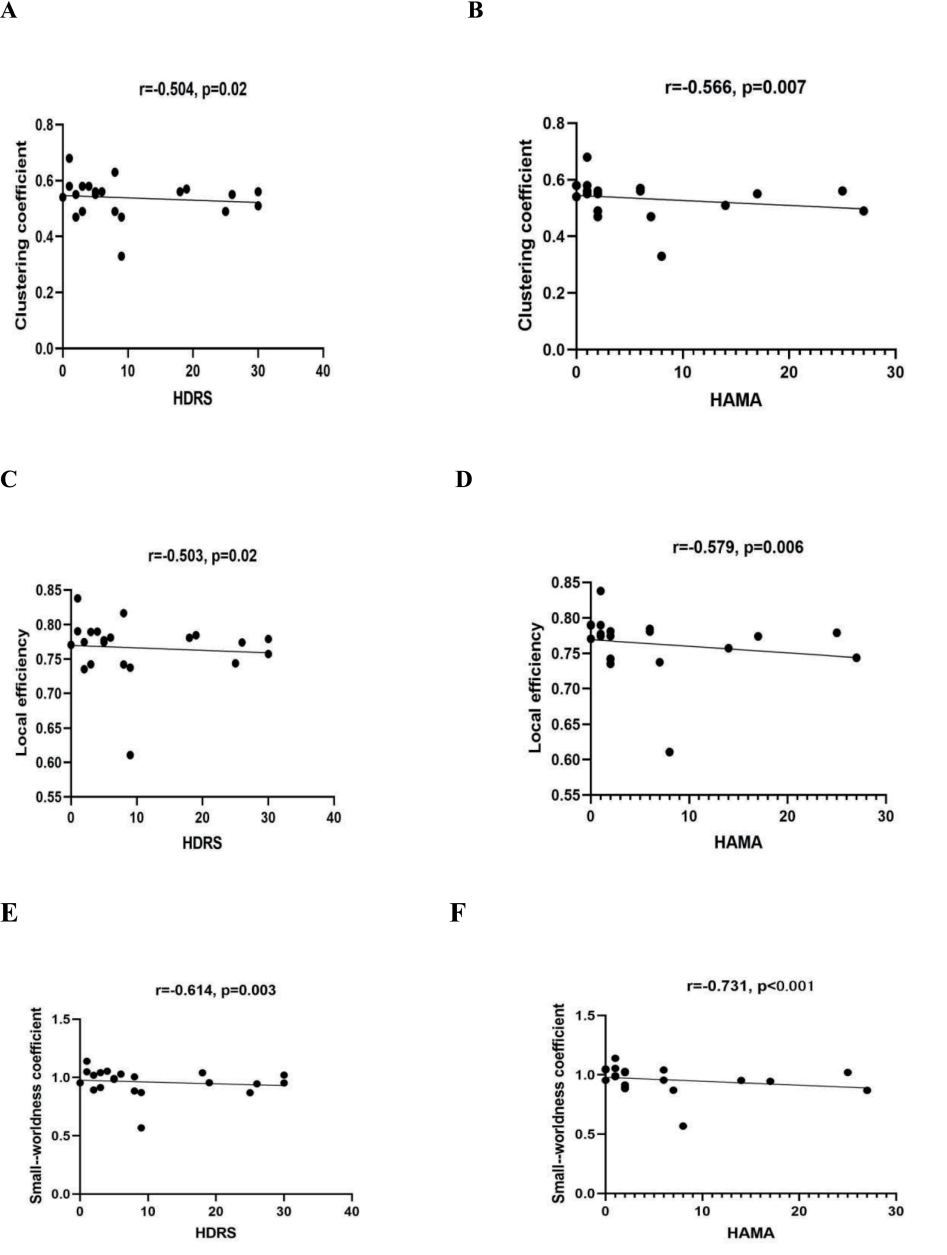
The correlation between HDRS and HAMA scores and network measures in the beta band of whole insular glioma patients. **(A,B)** represent the correlation between clustering coefficients and HDRS and HAMA scores, respectively; **(C,D)** represents the correlation between local efficiency and HDRS and HAMA scores, respectively; **(E,F)** represents the correlation between small-worldness coefficient and HDRS and HAMA scores, respectively.

**Figure 7:**
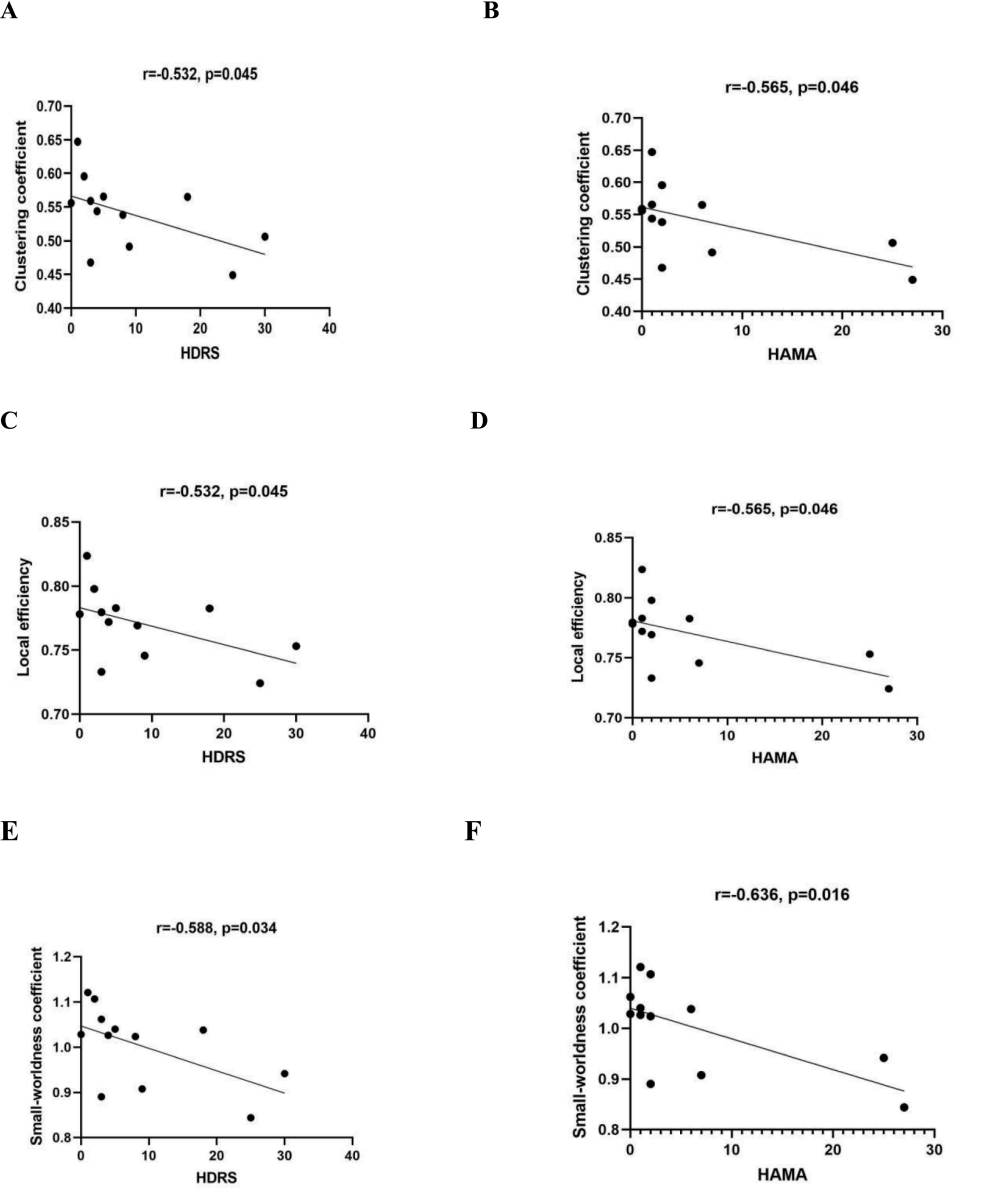
The relationships between HDRS and HAMA scores and network measures in the beta band of right insular glioma patients. **(A,B)** Represent the correlation between clustering coefficients and HDRS and HAMA scores, respectively; **(C,D)** represents the correlation between local efficiency and HDRS and HAMA scores, respectively; **(E,F)** represents the correlation between small-worldness coefficient and HDRS and HAMA scores, respectively.

Through assessment of the data distribution, we found that the neuropsychological test scores did not follow a normal distribution, so we used Spearman correlation analysis to explore the relationships between network measures and neuropsychological test scores in PT group. We found that it was only in the beta band under standard stimuli that a significant correlation was observed between network measures and neuropsychological test scores. Cp, locE, and the small-worldness coefficient all showed negative correlations with HDRS and HAMA scores. (Table 4, Figure 6). However, when we analyzed patients with left and right insula glioma separately, we found that only the Cp, locE, and the small-worldness coefficient of right insula glioma patients showed significant negative correlations with HDRS and HAMA scores in beta band under novel stimuli (Table 5, Figure 7). The Cp, locE, and the small-worldness coefficient of left insula glioma patients didn’t showed significant negative correlations with HDRS and HAMA scores (Table 6).

**Table 4:** Correlations between neuropsychological test scores and network metrics in the beta band.

Network measures	MoCA		MMSE		HDRS		HAMA	
	r	*p*	r	*p*	r	*p*	r	*p*
Clustering coefficient	0.194	0.400	−0.010	0.997	−0.504	0.020*	−0.566	0.007**
Local efficiency	0.223	0.330	−0.031	0.904	−0.503	0.020*	−0.579	0.006**
Small-worldness coefficient	0.325	0.150	−0.018	0.944	−0.614	0.030*	−0.731	<0.001**

*p < 0.05, **p < 0.01. Significant correlations are indicated in bold. ‘r’ denotes the Spearman correlation coefficient.

**Table 5:** Correlations between neuropsychological test scores and network metrics in the beta band of right insular glioma patients.

Network measures	MoCA		MMSE		HDRS		HAMA	
	r	*p*	r	*p*	r	*p*	r	*p*
Clustering coefficient	0.481	0.113	0.288	0.997	−0.532	0.045*	−0.565	0.046*
Local efficiency	0.481	0.113	0.288	0.904	−0.532	0.045*	−0.565	0.046*
Small-worldness coefficient	0.406	0.190	0.195	0.944	−0.588	0.034*	−0.636	0.016*

Significant correlations are indicated in bold. ‘r’ denotes the Spearman correlation coefficient. *p < 0.05.

**Table 6:** Correlations between neuropsychological test scores and network metrics in the beta band of left insular glioma patients.

Network measures	MoCA		MMSE		HDRS		HAMA	
	r	*p*	r	*p*	r	*p*	r	*p*
Clustering coefficient	0.252	0.513	0.150	0.749	−0.250	0.516	−0.403	0.282
Local efficiency	0.252	0.513	0.150	0.749	−0.250	0.516	−0.403	0.282
Small-worldness coefficient	0.454	0.220	−0.037	0.937	−0.433	0.244	−0.622	0.074

Significant correlations are indicated in bold. ‘r’ denotes the Spearman correlation coefficient.

## 4 Discussion

In the present study, we investigated differences in brain functional networks between patients with insular glioma and healthy controls during an auditory oddball task. We observed that, relative to healthy controls, the patient group exhibited significantly lower values for clustering coefficient (Cp), local efficiency (locE), and small-worldness in the beta frequency band under novel stimulus conditions. For right insular glioma patients, the Cp, locE, and the small-worldness were also lower than the healthy group within the beta band under novel stimuli. However, there was no significant differences of graph theory metrics between left insular glioma patients and healthy group within the beta band under novel stimuli. The Cp, locE, and the small-worldness coefficient of insular glioma patients all showed negative correlations with HDRS and HAMA scores at the beta band under standard stimulation. When we analyzed patients with left and right insula glioma separately, we found that only the Cp, locE, and the small-worldness coefficient of right insula glioma patients showed significant negative correlations with HDRS and HAMA scores in beta band under novel stimuli. The Cp, locE, and the small-worldness coefficient of left insula glioma patients didn’ t showed significant negative correlations with HDRS and HAMA scores.

The insula serves as a critical node within the core brain network, which is fundamental for sustaining neural activity during ongoing cognitive and behavioral tasks, and is thought to be implicated in target detection processes (4, 26, 27). The beta band is primarily associated with motor control, attention maintenance, cognitive processing, and sensory-motor integration (28–30). In the Oddball task, standard stimuli require sustained attention maintenance, while novel stimuli necessitate rapid information integration and response inhibition. The activity in the beta band may directly participate in the neural oscillatory synchronization processes of these advanced cognitive functions.

Our study found that there were 342 functional connectivities were significantly different at beta band under novel stimuli between insular glioma patients and healthy controls, while the number of significantly different functional connectivities at beta band under standard stimuli between the two groups are 220. This comparison indicates that under novel stimuli, there were more extensive functional connectivity abnormalities in the brain networks of patients with insular glioma and healthy controls, further confirming that novel stimuli posed a greater challenge to patients’ brain networks - novel stimuli require the brain to quickly mobilize more brain regions to participate in information integration, anomaly recognition, and response inhibition. However, the functional impairment of brain regions caused by gliomas makes it difficult to smoothly complete this complex neural coordination process, resulting in a significant increase in the number of functional connectivity differences.

Based on the analysis of graph theory indicators, the patient group showed a significant decrease in Cp, locE, and the small-worldness coefficient under novel stimuli, which was mutually confirmed by the widespread functional connectivity abnormalities. This once again confirmes that the information integration ability and response inhibition ability of patients with insular glioma had been severely impaired. This may be due to the fact that the insula, as a key hub of the brain network, not only destroys its direct functional connections with surrounding brain regions, but also indirectly affects the topological structure of the entire beta band network, leading to a decrease in local aggregation and information transmission efficiency. At the same time, the widespread abnormalities in functional connections further exacerbate the collaborative barriers between various brain regions. This kind of damage is reflected in clinical practice, which may lead to patients being unable to timely and accurately complete information recognition, integration, and response regulation when facing complex and changing environmental stimuli, thereby affecting their daily life self-care ability, attention concentration, and cognitive judgment function. This also provided a more intuitive quantitative basis for clinical assessment of patients’ cognitive impairment.

Further subgroup functional connectivity analysis was conducted by glioma side, and we found that this stimulus dependent functional connectivity difference also had significant lateral specificity: for left insular glioma patients, under novel stimuli, there were 764 functional connections with significant differences in the beta band between the patient group and the healthy control group, while under standard stimuli, the number of functional connections with significant differences in the beta band between the two groups was only 116, indicating that the degree of abnormality in brain network functional connectivity in left insular glioma patients significantly increased when faced with novel stimuli. For patients with right insula gliomas, under novel stimuli, there were 438 significant differences in beta band functional connections between the patient group and the healthy control group, while under standard stimuli, the number of significant differences in beta band functional connections between the two groups was only 18, indicating that the brain network of patients with right insula gliomas is more sensitive to new stimuli and functional connectivity abnormalities are more prominent.

However, when faced with novel stimuli, the patients group and the right insular glioma patients group still demonstrated significantly lower metrics for Cp, locE, and the small-worldness coefficient within the beta band compared with the healthy group. This may reflect the sensitivity of insular gliomas to specific stimulation conditions -as the core node of the brain’s salience network, the lesions in insular gliomas directly affect the brain’s ability to detect and process abnormal stimuli, and new stimuli have a higher cognitive load on the brain network, making it more susceptible to potential functional damage. This is because the impact of glioma on the destruction of brain neural network function is more prominent under high cognitive load conditions. This discovery provides a new research perspective for a deeper understanding of brain dysfunction in patients with glioma, indicating that the beta band graph theory index under novel stimuli may serve as a potential biological indicator for evaluating changes related to brain functional disorders, providing new ideas and basis for early identification and assessment of cognitive impairment in glioma patients in clinical practice.

No significant abnormalities were observed in the brain network topological metrics of patients with left insular glioma, and the core mechanism underlying this finding lies in the efficient interhemispheric compensatory plasticity of the left dominant hemisphere. As a core brain region associated with language and cognition, the left insula features a neural network with a high degree of functional specialization and neural connection redundancy. When the left insula was damaged by glioma, the brain prioritizes the activation of a transhemispheric compensatory mechanism, which compensates for the functional deficits of the left insula through the rapid reorganization of the right insula, prefrontal cognitive control network, and parietal lobe (31, 32). Consequently, no significant reductions were found in the integration and small-world properties of the whole brain network in these patients. In contrast, the right hemisphere serves as the core brain region for emotional perception and nonverbal communication, so patients with right insular glioma lack such an efficient compensatory mechanism following insular injury. Furthermore, as a core node of the brain’s salience network, lesions of the right insula directly impair the brain’s ability to detect and process novel stimuli, ultimately leading to significant abnormalities in brain network topological metrics in the beta band under novel stimuli. This hemispheric specific difference in plasticity constitutes the core neural mechanism underlying the regulation of abnormal brain network patterns by tumor lateralization in this study, and it further verifies the specificity and reliability of the primary findings of this research.

Our study also found that the Cp, locE, and the small-worldness coefficient of insular glioma patients all showed negative correlations with HDRS and HAMA scores at the beta band under standard stimulation. This means that although the patient’s brain maintains a relatively stable network activation pattern to some extent under standard stimulation, symptoms of depression and anxiety still have a significant impact on their brain network characteristics. Depressive symptoms may reduce brain excitability and affect the efficiency of information dissemination; anxiety symptoms may increase brain alertness, leading to excessive network activation, which is reflected in beta band graph theory indicators (33, 34). We found in the stratified analysis of insular gliomas on different sides that the Cp, locE, and the small-worldness coefficient of the right insular glioma patients also showed negative correlations with HDRS and HAMA scores at the beta band under standard stimulation. However, there was no significant negative correlations between neuropsychological test scores and network measures for left insular glioma patients. This may be due to the more prominent role of the right hemisphere in emotional perception, nonverbal communication, and social cognition. If the insular glioma in the right hemisphere disrupts this “emotion perception edge system” pathway, it may lead to increased sensitivity of patients to negative emotions, manifested as a stronger negative correlation between network measures and HDRS and HAMA scores. Besides, as mentioned earlier, the left insula belongs to the language and cognitive dominant hemisphere, and its damage triggers more active and efficient cross hemisphere compensation in the brain. The right insula and prefrontal networks quickly reorganize, replacing the emotional cognitive function of the left insula. Therefore, compared with the HC group, there was no significant difference in the network measures of left insular glioma patients, which may decreased the negative correlation between network measures and HDRS and HAMA scores of left insular glioma patients.

However, under the stimulation of novelty, there was no significant correlation between the graph theory indicators of beta band and the scores of HDRS and HAMA scales. This may be due to novel stimuli can widely activate multiple regions of the brain, triggering complex neural activity and making the relationship between emotions and brain networks more indirect and complex. In this case, depression and anxiety may affect the activity of brain networks through multiple pathways, and graph theory indicators of beta band may not fully reflect these complex effects. Therefore, under the stimulation of novelty, there was no significant correlation between the graph theory indicators of beta band and the scores of HDRS and HAMA scales.

In this study, tumor lateralization was incorporated as a core stratification factor into the full-process analysis of brain networks, aiming to clarify its potential impact on the primary finding that insular gliomas impair the integration and small-world properties of the whole brain functional network in the beta band under novel stimuli. Previous studies have confirmed that left and right insular gliomas can induce distinct patterns of brain network plasticity: as the dominant hemisphere for language and cognition, damage to the left insula triggers efficient interhemispheric compensatory plasticity, whereas the right insula, a core brain region for emotional perception, exhibits relatively limited brain network compensatory capacity following injury (31, 32). The findings of the present study strongly support this conclusion and further verify that tumor lateralization is not a confounding factor for the primary finding of this study, but rather a core characteristic and important supplement to it. In terms of the results, the primary finding of this study—the reductions in brain network Cp, locE, and the small-worldness coefficient in the beta band under novel stimuli, was mainly driven by patients with right insular gliomas. Owing to the interhemispheric compensatory plasticity of the left dominant hemisphere, patients with left insular gliomas showed no significant abnormalities in brain network topological metrics. This result does not negate the primary finding; instead, it defines the hemispheric specific boundary of the primary finding, namely that the impairing effect of insular gliomas on the beta band brain network is predominantly concentrated in populations with right insular lesions.

However, this study has several limitations. Firstly, the small sample size weakened the statistical power of the current analysis; second, although this study found a negative correlations between Cp, locE, and the small-worldness coefficient and HDRS and HAMA scores at the beta band under standard stimulation, it is currently not possible to directly use them as biomarkers for predicting patients’ anxiety and depression emotions. However, this correlation provides important clues for further exploring the relationship between emotions and brain neural networks. Subsequent research can expand the sample size and delve deeper into the neural mechanisms behind this negative correlation; third, due to our inability to obtain MRI data of patients, therefore, we used the Colin27 MNI template for normalization and reconstruction of the sources generating the EEG signal. However, brain lesions caused by gliomas can significantly alter the anatomical geometry and conduction of the EEG signal, so the use of a model based on healthy subjects may not be reliable. In future research, we will collect more patients and attempt to conduct fMRI analysis; last but not the least, owing to our limited sample size and because patients with insular glioma are mainly of lower grade, the distribution of glioma grades in the patient group in our study was uneven. In our study, there were 31 patients with lower grade gliomas (WHO grade 2–3), including 21 patients with WHO grade 2, 10 patients with WHO grade 3, and 2 patients with high-grade gliomas (WHO grade 4), so we did not made a stratified exploratory analysis. In future research, we will collect more patients and attempt to conduct stratified analysis.

In summary, for insular glioma patients, especially right insular glioma patients, the differences of Cp, locE, and the small-worldness coefficient in beta band under novel stimuli may provide new potential indicators for disease assessment. By detecting these characteristic changes in the brain neural network of patients when faced with novel stimuli, it helps to more accurately identify disease states and provide a basis for the development of clinical treatment plans. Although the negative correlation between graph theory indicators and emotion scale scores under standard stimuli cannot be directly used as a biomarker for predicting patients’ anxiety and depression, it offers new perspectives for elucidating the neural mechanisms of emotional impairments in insular glioma patients. This implies that neurosurgeons should consider the influence of patients’ emotional status on brain network function, in addition to focusing on the treatment of insular gliomas, and take comprehensive intervention measures to improve patients’ emotions and quality of life. In addition, this study enriches our understanding of the dynamic changes in neural networks of patients with insular gliomas, revealing the complexity and diversity of brain compensation mechanisms under different stimulation conditions. This will help further improve the theoretical model of the brain neural network and provide important references and inspirations for subsequent related research.

## 5 Conclusion

Compared with healthy controls, patients with insular gliomas exhibited significant impairments in beta band brain network function under novel stimuli, characterized by reduced Cp, locE, and small-worldness coefficient. Notably, this network impairment showed pronounced lateralization: right insular glioma patients displayed marked reductions in the above beta band topological indicators, while left insular glioma patients showed no significant differences from healthy controls. This hemisphere specific difference is likely attributed to the efficient interhemispheric compensatory plasticity of the left hemisphere (the dominant hemisphere for language and cognition), which can reorganize neural networks to offset functional deficits caused by left insular lesions.

Correlation analysis further demonstrated that the beta band Cp, locE, and small-worldness coefficient of insular glioma patients were negatively correlated with HAMA and HDRS scores under standard auditory stimuli. This indicates that anxiety and depressive symptoms exert a significant impact on brain network characteristics even when the brain maintains relatively stable activation patterns. Stratified analysis highlighted that this negative correlation was specific to right insular glioma patients, whereas left insular glioma patients showed no such significant association, which consistented with the stronger role of the right hemisphere in emotional perception and nonverbal emotional processing, and the effective compensation for emotional cognitive functions in the left hemisphere after injury.

Collectively, the beta band Cp, locE, and small-worldness coefficient under novel stimuli emerge as potential neurophysiological indicators for the disease assessment of insular gliomas, particularly for right insular lesions. These indicators can objectively reflect the extent of brain network impairment and complement clinical evaluations. Additionally, the significant association between emotional states and brain network properties in right insular glioma patients emphasizes the necessity of integrating emotional intervention into clinical management. Neurosurgeons should not only focus on tumor resection and pathological treatment but also pay close attention to patients’anxiety and depressive symptoms, implementing comprehensive interventions to improve emotional well-being and overall quality of life.

## Author’s note

All authors confirm that the following manuscript is a transparent and honest account of the reported research. This research is related to a previous study by the same authors titled *“The correlation between auditory event-related potentials characteristics and cognitive function in insular glioma patients.”* The previous study was performed on the characteristics of auditory event-related potentials (AERP) and event-related spectral perturbation (ERSP) in patients with insular gliomas, and the current submission is focusing on constructing functional brain networks for patients with insular gliomas using task-based electroencephalography data, and investigating the effects of insular gliomas and emotional status on brain network organization. The study is following the methodology explained in the task-based EEG data acquisition procedure.

## Data Availability

The raw data supporting the conclusions of this article will be made available by the authors, without undue reservation.
